# Sedation depth and mortality in mechanically ventilated critically ill adults

**DOI:** 10.1186/cc10930

**Published:** 2012-03-20

**Authors:** Y Shehabi, S Kadiman, L Chan, W Ismail, M Saman, A Alias

**Affiliations:** 1 University New South Wales, Randwick, Australia; 2 National Heart Institute, Kuala Lumpur, Malaysia; 3 University Malaya, Kuala Lumpur, Malaysia; 4 Raja Perempuan Zainab II Hospital, Kota Bharu, Malaysia; 5 Sarawak General Hospital, Kuching, Malaysia; 6 Malacca General Hospital, Malacca, Malaysia

## Introduction

Deep sedation is common in ventilated patients, particularly in the first 48 hours in the ICU, which may adversely affect outcomes such as mortality. This period is usually unobserved in clinical trials due to late randomisation. We investigated the relationship between early sedation depth, sum of Richmond Agitation Sedation Scale (RASS) -3 to -5 and clinical outcomes, including mortality.

## Methods

A waiver of consent was granted. In collaboration with the Australian New Zealand Intensive Care Research Centre, we conducted a multicentre prospective longitudinal cohort study in 11 centers in Malaysia. Critically ill patients ventilated and sedated ≥24 hours were followed from ICU admission to hospital discharge. The administration of all sedatives was measured daily. Four-hourly RASS assessments were conducted and delirium assessed daily (CAM-ICU during light sedation RASS -2 to +1). Multivariable Cox regression proportional hazard was used to quantify relationships between early deep sedation and time to extubation and delirium occurring after 48 hours and hospital mortality adjusting for diagnosis, age, gender, APACHE II score, operative, elective, early use of vasopressors and dialysis.

## Results

We studied 259 patients with mean (SD) age 53.1 (15.9) years and APACHE II score 21.3 (8.2), ventilated for median (IQR) 5 (3 to 8.8) days. Hospital mortality was 82 (31.7%). Midazolam and morphine were the commonest agents used, given to 241 (93.1%) and 201 (77.6%) patients respectively. Over 2,657 study days, 13,836 assessments were conducted. Deep sedation was recorded in 187 (72%) patients within 4 hours of commencing ventilation and in 159 (61%) patients at 48 hours. Daily interruption was used on 20% of study days. Delirium occurred in 114 (43%) of assessed patients with a mean (SD) duration of 1.3 (2.2) days. Early deep sedation independently predicted time to hospital death (HR 1.11, 95% CI 1.05 to 1.18, *P *< 0.001) and time to extubation (HR 0.93, 95% CI 0.89 to 0.96, *P *= 0.001) but not time to delirium occurring after 48 hours (HR 0.98, 95% CI 0.93 to 1.03, *P *= 0.46). Midazolam cumulative dose in the first 48 hours was significantly associated with the number of RASS assessments ≤-3 (*P *< 0.001).

## Conclusion

Early ICU sedation depth is a modifiable risk factor for delayed extubation and increased risk of death and should be considered in future sedation trials.
